# Perception of consanguineous marriage among the qatari population

**DOI:** 10.3389/fpubh.2023.1228010

**Published:** 2023-08-04

**Authors:** Yasamin Abdu, Khalid Ahmed, Mohamed Izham Mohamed Ibrahim, Mariam Abdou, Arwa Ali, Hind Alsiddig, Nagah A. Selim, Mohammed A. Yassin

**Affiliations:** ^1^Community Medicine Department, Hamad Medical Corporation, Doha, Qatar; ^2^Department of Hematology, NCCCR, Hamad Medical Corporation, Doha, Qatar; ^3^College of Pharmacy, QU Health, Qatar University, Doha, Qatar; ^4^College of Medicine and Surgery, University of Bahri, Khartoum, Sudan; ^5^Nile University, Khartoum, Sudan; ^6^Community Medicine Department, Primary Health Care Corporation, Doha, Qatar

**Keywords:** consanguineous marriage, consanguinity, perception, premarital screening, genetic diseases, sickle cell disease, thalassemia, genetic blood diseases

## Abstract

**Background:**

Hereditary blood diseases are widespread among the Arab population due to the high rates of consanguineous marriages; research regarding the perception of consanguineous marriage in some countries, such as Qatar, is extremely scarce. Therefore, this study aimed to investigate the prevalence of consanguineous marriage and assess the perception of consanguineous marriage among the Qatari population.

**Methods:**

A cross-sectional study used a self-administered questionnaire among 395 Qatari adults aged 18–35 who attended primary healthcare institutions in Qatar. A convenience sampling technique was used to select the study participants. An independent *t*-test was used to compare the significance of the mean between the two groups with positive and negative perceptions of consanguineous marriage. Categorical data were analyzed for association using the chi-square or Fisher's exact test. Finally, a multiple logistic regression analysis was conducted to determine the significant predictors of the positive perception of consanguineous marriage. A significant level was set at *p* < 0.05.

**Results:**

Approximately 45% of the participants had a positive perception toward consanguineous marriage, and the most common reason stated by those participants was “habit and traditions.” The prevalence of consanguineous marriage among married couples was 62.6%, and among those with consanguineous marriage, most were married to first cousins (81.7%). Moreover, compared to the participants with negative perceptions of consanguineous marriage, those with positive ones were significantly older, married, with lower educational levels and higher monthly income levels, did not hear about glucose-6-phosphate dehydrogenase (G6PD) deficiency, did not know what kinds of diseases are being screened in the premarital test, and were married to a relative.

**Conclusion:**

The prevalence of consanguineous marriage is high among the Qatari population, and this requires an immediate need for community-based campaigns to raise public awareness about the problem and its potential impact.

## 1. Introduction

The Premarital Genetic Screening Program Services implemented in Qatar in December 2009 are available at eight primary health centers; promoting the health and couples is primarily a preventive approach, mandatory for legally certified marriages, as in many Arab countries (1, 2). Besides premarital screening tests for infectious diseases, such as hepatitis B, hepatitis C, HIV, and syphilis and checking immunity against measles, this program involves screening for hemoglobinopathies, namely sickle cell disease (SCD) and thalassemia, as well as other genetic disorders, including homocystinuria, cystic fibrosis, and spinal muscular atrophy, and the results were obtained through blood tests (3).

Regarding screening for sickle cell traits and thalassemia as a part of the premarital screening program, both couples receive health education about hereditary disorders. They are subjected to a blood test, and if they are found to be carriers, then accordingly, they are referred to the hematological department and genetic counselling, where they are counselled about the consequences of hereditary disorders in future offspring and the available technologies such as *in vitro* fertilization (IVF) that can assist them in birthing unaffected children (1).

Even though there has been a mandatory premarital genetic screening program in Qatar since 2009, the incidence and prevalence of SCD and thalassemia are still high in the country, most probably due to the high rate of consanguineous marriage. Consanguineous marriage is a marriage between second cousins or closer (2). The prominent harm of consanguineous marriages is the higher frequency and incidence of autosomal recessive disorders and higher morbidity and mortality rates among the offspring (3).

Sickle cell disease (SCD) and thalassemia are among the significant health problems in Arabic countries, particularly in Qatar (4, 5). Sickle cell disease (SCD) is one of the most common genetic blood diseases inherited as an autosomal recessive blood disorder, most common among people from Africa, India, the Caribbean, the Middle East, and the Mediterranean (6). The high prevalence of SCD in Arab countries is due to many factors, such as the high rate of consanguineous marriage between first cousins, which is more than 50% of total marriages in most Arab countries, including Qatar (7). Additionally, SCD carriers are resistant to Falciparum malaria, which is endemic in the region (8). Moreover, there is a lack of awareness about inherited hematological diseases among the Arab population (9).

In Qatar, there are approximately 550 Qatari patients with SCD, and most of the cases are distributed in the Northern Province and Doha, the capital of Qatar (10). Moreover, in a survey that was conducted among 1,702 Qataris, they found that sickle cell hemoglobin (Hb-s) was found in 14.63% of those tested, and 0.76% had β-thalassemia major (11).

Thalassemia is a heterogeneous grouping of genetic disorders that result from a decreased synthesis of alpha or beta chains of hemoglobin (12). There are two types of thalassemia, depending on which kind of globin gene is mutated: alpha- (α-) thalassemia and beta- (β-) thalassemia. α-Thalassemia occurs when one or more of the four α-globin genes are damaged or altered, while β-thalassemia occurs when both β-globin genes are damaged or mutated (13, 14). Moreover, thalassemia major occurs when a child inherits two defective globin genes, one from each parent, and thalassemia minor occurs when the child inherits one defective globin gene from only one parent [15.] Thalassemia is inherited as an autosomal recessive trait; thus, consanguineous marriage is one of the most common risk factors of thalassemia, as the possibility of both parents being carriers is high.

A study that was conducted among 1,800 Qatari in 2004 estimated a consanguineous marriage rate of 54% (16). Furthermore, another study (17) that was conducted in 2012 to assess the effect of consanguineous marriage in a cohort of patients with specific genetic disorders found that all consanguineous marriages had a significantly higher risk of autosomal recessive disorders than non-consanguineous marriages. Therefore, in this study, we aimed to estimate the prevalence of consanguineous marriage and assess the perception of consanguineous marriage among the Qatari population. Furthermore, our results will help develop a clear public health message to address the problem of the high rate of consanguineous marriage in the country.

## 2. Methods

### 2.1. Study design and study setting

This analytical cross-sectional study was conducted between October 2022 and December 2022 at three primary health centers under the Primary Health Care Corporation. The health services provided in those health centers are accessible to the entire registered Qatari population. Moreover, the three health centers are in different geographical areas and cover the Qatari population's different socioeconomic classes and educational backgrounds.

### 2.2. Study procedure

The target population included Qatari men and women who attended Primary Health Centers for any reason during the data collection period, were willing to participate, and fulfilled the eligibility criteria.

Our inclusion criteria included Qatari men and women of all educational levels, married or unmarried, aged 18 to 35 (the age-range selection criterion has been set to reflect the expected marital age in Qatar), who agreed to sign the written informed consent, and who were able to read and write either Arabic or English. In addition, we excluded adults with cognitive impairment that interfered with communication. The ethics approval was obtained from the Institutional Review Board (IRB) of the Primary Health Care Corporation (PHCC/DCR/2022/06/032).

The previous prevalence of consanguineous marriage in Qatar of 54% (15) was utilized to calculate the sample size with a 5% degree of precision and 95% confidence limits. Accordingly, the sample size of 381 was calculated using the following formula (18):


$$\begin{array}{l} n=\frac{\left[{\left([Z\;1-\alpha /2)\right]}_{x}px(1-p)\right]}{d2} \end{array}$$

Moreover, we added 20% of the computed sample size as the expected non-response rate; hence, the estimated sample size was 457.

A multistage sampling technique was used. First, a cluster sampling technique was utilized to randomly select three health centers primarily for the Qatari population with a high attendance rate. Second, a convenience sampling technique was used to choose adults aged 18 to 35 of Qatari nationality and attending any clinic services in the selected primary health centers. An equal number of participants were selected from each health center (152 participants were approached from each health center). Finally, the selected participants were interviewed, and the orientation of the patients about the study was carried out before obtaining written informed consent with an emphasis on the subject's right to refuse participation.

#### 2.2.1. Study instrument

A structured self-administered questionnaire was developed after reviewing the relevant literature. The content and face validity were established through an extensive literature review and consultation with experts in the field of community medicine and hematology. The format of the questions varied between multiple-choice, dichotomous questions (yes/no), and open-ended questions. The questionnaire was initially prepared in English and translated into Arabic with back translation by a bilingual translator.

Before data collection, a pilot study was conducted among a convenience sample of 20 participants from one primary health center to pre-test the comprehensibility and appropriateness of the questionnaire. In addition, the pilot phase provided an opportunity to measure the time needed to complete each questionnaire by the participants. As a result, some necessary adjustments were made to the questionnaire. The piloted 20 participants were omitted from the study database.

#### 2.2.2. Independent variables

The independent variables included age in years, gender of the participant, marital status, educational status, occupation, monthly family income in Qatari Riyal (QAR), personal and family history of SCD or sickle cell trait, and personal and family history of other genetic blood diseases such as thalassemia or G6PD deficiency. In addition, participants' awareness of inherited genetic blood diseases and premarital screening tests were assessed by questions such as “Have you heard about SCD, thalassemia, and G6PD deficiency?” “Is there a treatment for genetic blood diseases?” “Do you think the family suffers from psychological pressure if one of its members suffers from one of the genetic blood diseases?”, “What diseases do we screen for in the premarital screening?” and “Do you think that premarital screening limits the spread of genetic diseases?”

#### 2.2.3. Outcome variables

Consanguineous marriage was assessed using the yes/no question. Then, the degree of consanguineous marriage was evaluated as an ordinal variable, and it included first cousin (a child of a parent's sibling; a nephew or niece of a parent; a child of one's uncle or aunt), first cousin once removed (parents' first cousin or first cousin's child), and second cousin (the child of a parent's cousin) (16). Perception of consanguineous marriage was assessed by the question “Do you prefer to marry a relative?” using the yes/no question. Those who answered “yes” were considered to have a positive perception of consanguineous marriage, and those who answered “no” were considered to have a negative perception. The reason why the participants preferred or did not prefer consanguinity was assessed using an open-ended question.

### 2.3. Statistical analysis

The Excel and IBM SPSS statistical programs (v 29, Armonk, NY: IBM Corp) were used to manage and analyze the data. First, continuous data were tested for normality. Second, an independent *t*-test was used to compare the significance of the mean between the two groups with positive and negative perceptions of consanguineous marriage. Moreover, categorical data were analyzed for association using the chi-square or Fisher's exact test. Finally, a multiple logistic regression analysis was conducted to determine the significant predictors of the positive perception of consanguineous marriage. A significant level was set at *p* < 0.05.

## 3. Results

### 3.1. Background characteristics

During the data collection period (October 2022–December 2022), 475 adults with Qatari nationality were approached to participate in the study. Approximately 62 refused to participate, and 395 eligible participants were included in the study, with a response rate of 83.1%. The participant's mean age (±SD) was 27.4 (±6.0) years. Most of the study participants (81%) were women (*n* = 320), 63% were married (*n* = 249), and 35.9% held a university degree or higher education (*n* = 142). In addition, 51.4% of the participants were currently employed (*n* = 203). The majority of the participants (87.6%) had a monthly family income of more than 10,000 QR.

In our sample, eight participants (2%) were SCD patients, and six participants (1.5%) had other genetic blood diseases such as thalassemia and glucose-6-phosphate dehydrogenase deficiency. Moreover, 20 participants (5.1%) had a family history of SCD, and 32 participants (8.1%) had a family history of other genetic blood diseases.

### 3.2. Prevalence and perception of consanguineous marriage

In our sample, 45.1 % of the participants positively perceived consanguinity marriage and answered “yes” to the question, “Do you prefer to marry a relative?”. The most common reasons stated by those who preferred consanguineous marriage are “habits and traditions,” followed by “more comparability and understanding between relatives,” and “premarital test is enough to reduce the risk of the disease,” 38.2%, 28.1%, and 2.7%, respectively (see Table 1). On the other hand, the participants who did not prefer consanguineous marriage stated that it is mostly “to avoid genetic disease and have a healthy child” (80.6%) and “to avoid family problems” (14.3%).

**Table 1:** Distribution of sample by the perceptions about consanguineous marriages.

**Indicators and variables**	**Frequency**	**Percentage (%)**
**1. Perception and prevalence of consanguineous marriage**		
Preferring to marry a relative (positive perception of consanguineous marriage)	178	45.1%
Reasons behind positive perception of consanguineous marriage:		
“Premarital test reduces the risk of genetic disease by detecting any problem.”	48	27%
“Habits and traditions”	68	38.2%
“Comparability and understanding in consanguineous marriage”	50	28.1%
“Strengthening family relationship.”	9	5.1%
“Genetic disease-free healthier children.”	2	1.1%
No reason	1	0.6%
Reasons behind negative perception of consanguineous marriage:		
“Habits and traditions”	4	1.8%
“Comparability and understanding between relatives in non-consanguinity marriage”	4	1.8%
“Genetic disease-free healthier children.”	175	80.6%
“Fewer family problems.”	31	14.3%
No reason	3	1.4%
Prevalence (practice) of consanguineous marriage	154	62.6%
Degree of consanguinity among married couples:		
First cousin	125	81.7%
The first cousin once removed	22	14.4%
Second cousin	6	3.9%
Premarital screening test results might lead to a marriage cancelation	351	88.9%
**2. Awareness about inherited genetic blood diseases and premarital screening tests**		
Thalassemia		
Sickle cell disease	60	15.2%
G6PD deficiency	130	32.9%
Presence of treatment for the inherited genetic blood diseases	75	19.0%
Psychological pressures in the family as a result of inherited genetic	36	9.1%
blood diseases	323	81.8%
Tests that are done before marriage	163	41.4%
Correctly identified the diseases being screened	22	5.6%
Screening helps to limit genetic diseases	370	93.7%

%, percentage; G6PD deficiency, glucose-6-phosphate dehydrogenase deficiency.

**Figure 1:**
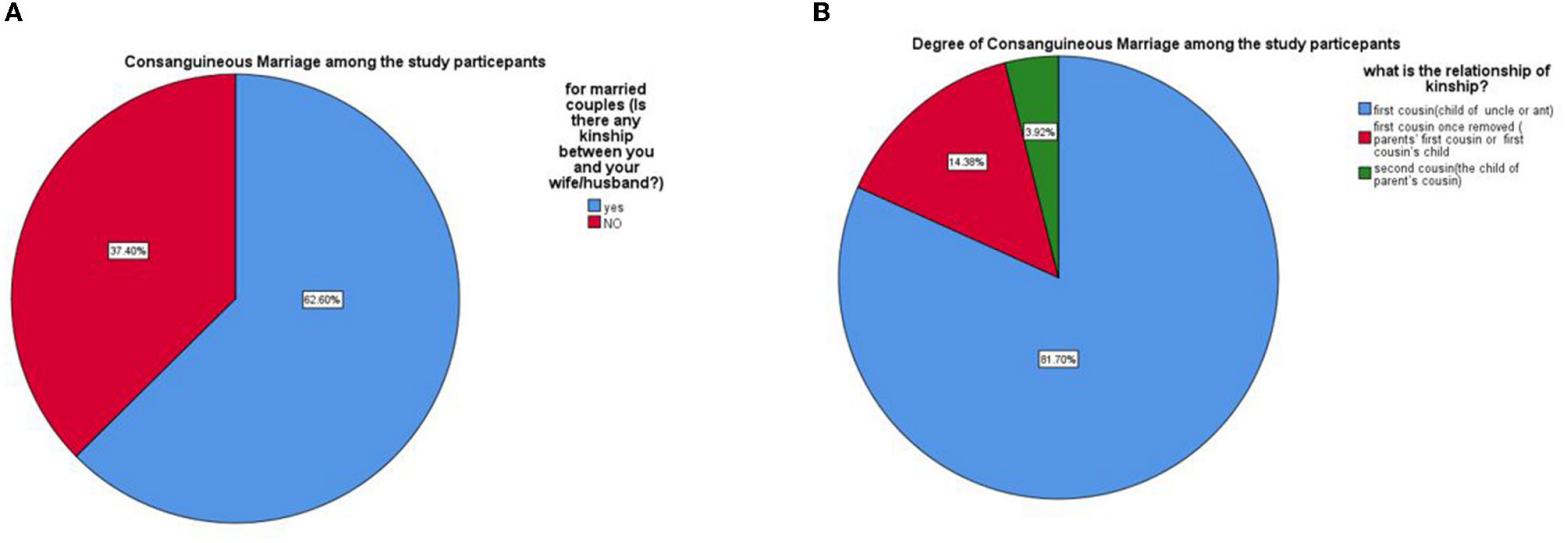
**(A)** Consanguineous marriage among the Qatari population. **(B)** The degree of consanguineous marriage among the Qatari population.

The prevalence of consanguineous marriage among married couples was 62.6%, as seen in Figure 1A. Moreover, among those with consanguineous marriage, the majority were first cousin marriages (81.7%), followed by marriage to a first cousin once removed (14.8%), as shown in Figure 1B.

When the study participants were asked about whether the test result of the premarital screening affected their decision to marry the other person, most of the participants (88.9%) answered yes; however, a minority of them (11.1%) said that they would still marry the other partner regardless of the test result, as seen in Table 1.

### 3.3. Participants' awareness of inherited genetic blood diseases and premarital screening tests

In our sample, most participants did not hear about thalassemia, sickle cell disease, or G6PD deficiency, accounting for 84.8%, 67.1%, and 81%, respectively. Moreover, most study participants (64.6%) did not know whether there was a treatment for inherited blood diseases. Most of the participants (81.8%) thought that the family would suffer from psychological stress if one of its members suffered from one of the genetic blood diseases. Although 41.4% of the participants claimed they knew about the tests on those about to get married, only 5.6% knew the diseases screened for in the premarital screening. On the other hand, almost all the participants (93.7%) agreed that premarital screening limits the spread of genetic diseases, as shown in Table 1.

### 3.4. Determinant of the perception of consanguineous marriage

With regard to the background characteristics, a statistically significant difference (*p*-value < 0.05) was found between those with consanguineous marriage (positive perception) and those against it (negative perception) in terms of age, marital status, educational level, and monthly family income.

The median age (±SD) of those who preferred consanguineous marriage was 29.1 (5.8), and the median age for their counterparts who did not prefer consanguineous weddings was 26.2 (5.9) (*P*-value < 0.001). Moreover, most of the married participants preferred consanguineous marriage (67.4%); however, most of the single participants did not prefer consanguineous marriage (52.1%) (*P*-value < 0.001).

Furthermore, participants with educational levels up to primary school preferred consanguineous marriage compared with their counterparts with higher education qualifications (21.9% vs. 9.2%, respectively). On the other hand, those with higher levels of education chose non-consanguineous marriage (9.8% vs. 78.1%), with a *P*-value of 0.003. Additionally, as the monthly family income increased (≥10.000 QR), the participants were more inclined to prefer consanguineous marriage, with a *P*-value of 0.013. In addition, all the study participants with SCD (eight participants) preferred non-consanguineous marriage, as shown in Table 2.

**Table 2:** Association between indicators and perceptions about consanguineous marriages.

**Items**	**Perception of consanguineous marriage**		**P-value**
	**Positive perception**	**Negative perception**	
	**Frequency (%)**	**Frequency (%)**	
**1. Background variables**			
**Age:**			
Mean (SD)	29.1 (5.8)	26.2 (5.9)	**<0.001**
**Gender**			
Male	35 (19.7)	40 (18.4)	0.757
Female	143 (80.3)	177 (81.6)	
**Educational level**			**0.003**
Up to primary school	39 (21.9)	20 (9.2)	0.758
Secondary school and higher education	139(78.1)	197 (90.8)	
**Employment**			**0.013**
Yes	93 (52.2)	110 (50.7)	
No	85 (47.8)	107 (49.3)	
**Monthly income (QAR)**			0.168
Up to 10,000	14 (7.9)	35 (16.1)	
More than 10,000	164 (92.1)	182 (83.9)	
**Family history of sickle cell anemia?**			
Yes	12 (6.7)	8 (3.7)	
No	166 (93.3)	209 (96.3)	
**Family history of other genetic blood diseases, such as thalassemia and G6PD deficiency**			0.180
Yes	18 (10.1)	14 (6.5)	
No	145 (81.5)	175 (80.6)	
Do not know	15 (8.4)	28 (12.9)	
**2. Awareness of inherited genetic blood diseases and premarital screening tests**			
**Thalassemia**			
Yes	26 (14.6)	34 (15.7)	0.770
No	152 (85.4)	183 (84.3)	
**Sickle cell disease**			0.324
Yes	54 (30.3)	76 (35.0)	
No	124 (69.7)	141 (65.0)	
**G6PD deficiency**			**0.005**
Yes	23 (12.9)	52 (24.0)	
No	155 (87.1)	165 (76.0)	
**Presence of treatment for genetic blood diseases**			0.128
Yes	13 (7.3)	23 (10.6)	
No	55 (30.9)	49 (22.6)	
Do not know	110 (61.8)	145 (66.8)	
**Psychological pressures in the family as a result of inherited genetic blood diseases**			
Yes	148 (83.1)	175 (80.6)	0.601
No	30 (16.9)	42 (19.4)	0.05
**Tests that are done before marriage**			
Yes	70 (39.3)	94 (43.3)	0.175
No	108 (60.7)	123 (56.7)	
**Correctly identified the diseases being screened**.			
Yes	5 (7.1%)	17(17.5%)	
No	65(92.9%)	80(82.5%)	
**Screening helps to limit genetic diseases**			
Yes	170 (95.5)	200 (92.2)	
No	8 (4.5)	17 (7.8)	
**3. Practice of consanguineous marriage**			
**Consanguineous marriage**			
Yes	110 (82.7)	50 (44.2)	**0.001**
No	23 (17.3)	63 (55.8)	
**Degree of consanguineous marriage**			
First cousin	90 (81.8)	35 (70.0)	0.104
Others	20 (18.2)	15 (30.0)	
**Test results might lead to wedding cancelation**			
Yes	160 (89.9)	206 (94.9)	0.056
No	18 (10.1)	11 (5.1)	

%, percentage; SD, standard deviation; QAR, Qatari Riyal, G6PD deficiency, glucose-6-phosphate dehydrogenase deficiency.

Moreover, most study participants who preferred consanguineous marriage did not hear about G6PD deficiency (78.1%) or know what diseases are being screened in the premarital test (92.9%), with a *P*-value of 0.005 and 0.05, respectively, as shown in Table 2.

Furthermore, most of the participants who preferred consanguineous marriage were married to a relative (82.7% vs. 17.3%) with a *p*-value of **<**0.001, as shown in Table 2.

### 3.5. Predictors of the positive perception of consanguineous marriage

The parameters that showed significance in the bivariate analysis were used in the multiple logistic regression analysis to determine the predictors for the patient's preference to marry a relative. Using the Hosmer–Lemeshow test, *P*-value = 0.324, i.e., the regression model indicated no evidence of poor fit (i.e., it is a good fit). However, after adjusting for the other variables, only four parameters have proven to be significant predictors for patients' preference to marry a relative: age (*p*-value = 0.023), monthly family income (*p*-value = 0.032), if patients have not heard about G6PD deficiency (*p* < 0.001), and if the patients have consanguinity with their spouse (*p* < 0.001).

Relatively older participants (>29 years) were more likely to have a positive perception of consanguineous marriage (adjusted OR = 1.082, 95% CI: 1.011–1.157). Moreover, those with higher monthly family income (≥10.000QR) were approximately three times more likely to prefer consanguineous marriage than their counterparts with lower monthly payments (aOR = 3.039, 95% CI: 1.101–8.384). On the other hand, patients who have heard about G6PD deficiency were approximately 72% less likely to have a positive perception of consanguineous marriage than their counterparts who have not heard about G6PD deficiency (aOR = 0.282, 95% CI: 0.134–0.597). Finally, patients with any kinship between them and their spouses were nearly seven times more likely to have a positive perception of consanguineous marriage (aOR = 6.739, 95% CI: 3.560–12.759), as shown in Table 3.

**Table 3:** Predictors of the perception of consanguinity marriage among the Qatari population.

**Explanatory variables**	**Preference to marry a relative**		**P-values**
	**Adjusted odds ratio (AOR)**	**95% CI Of (AOR)**	
Age	1.082	1.011–1.157	0.023
Marital status	0.638	0.239–1.705	0.370
Educational level	0.807	0.428–1.519	0.506
Monthly family income	3.039	1.101–8.384	0.032
Have you heard about G6PD deficiency?	0.282	0.134–0.597	<0.001
For married couples (Is there any kinship between you and your wife/husband?	6.739	3.560–12.759	<0.001
Constant	0.018		<0.001

AOR, adjusted odds ratio; G6PD deficiency, glucose-6-phosphate dehydrogenase deficiency.

## 4. Discussion

This study investigated the perception of consanguineous marriage among the adult Qatari population in 2022. Approximately 45% of the study participants preferred consanguineous marriage over non-consanguineous marriage, and 62.6% were married to a close relative. A significant association was found between a positive perception of consanguineous marriage and the participant's age, marital status, educational level, monthly family income, and being a patient with SCD. Moreover, participants who positively perceived consanguineous marriage did not hear about G6PD deficiency or the diseases screened for in the premarital test.

Our study showed that the prevalence of consanguineous marriage among the adult Qatari population had increased significantly since 2004; a population-based study conducted in Qatar estimated a prevalence of 54% (16) ^.^The rate of consanguineous marriage in Qatar is higher than observed in most Arab countries, including Oman (52%) (19), Kuwait (54.3%) (20), Saudi Arabia (57%) (21), and Sudan (63.3%) (22). One possible explanation is that recent rapid growth and development in Qatar have made Qataris feel less secure; therefore, they became more reluctant for potential spouses outside their families (23). Similar to the previous study in the country, there was a predominance of first-cousin unions among the consanguineously married couples, and this finding is in line with the pattern of consanguineous marriage in the Middle East countries such as Turkey (24) and Saudi Arabia (25). There are many reasons why first-cousin unions are culturally preferred. As stated by our study participants, the most important reasons were ”tradition and habits within their families,“ as marriage in many Arab countries is regarded as a family decision and not just the couple's decision, and a better relationship with a spouse that is well known and part of the extended family (26). Similar reasons were mentioned by the Qatari participants in a survey conducted in 2007 (27).

This study observed various patterns of consanguineous marriage in Qatar, some of which are common in other Arab countries. For example, those who are young with a lower level of education are more likely to marry their biological relative, but, again, these patterns are common in other Arab countries (28–30). In addition, we found that participants with higher monthly family incomes preferred to marry a relative. A possible reason is the lesser cost of arranging the marriage and preserving the wealth within the family (31).

Like our findings, a study conducted in Oman (11) found that the majority (72.4%) of the single participants chose to avoid consanguineous unions to prevent the chance of developing hereditary diseases (79%) and the remainder chose to avoid family problems (8%). Moreover, the awareness of SCD among our study participants was more than that of thalassemia and G6PD deficiency. However, in a later study (9), the participant's understanding of SCD and G6PD deficiency was reasonably high. On the other hand, approximately half of the participants reported awareness of thalassemia, which is likely due to the country's differential efforts in health education.

Similarly, in an Omani study (9), the majority reported that they were aware that hereditary blood disorders could carry significant psychological burdens on families. Similarly, most of the participants in the later study needed to be made aware of premarital testing. In contrast, in a study conducted in Saudi Arabia (33), the participants had good knowledge about the nature of the tests; in fact, 94% of the respondents knew that genetic disorders were the target for conducting the test.

Many individuals choose to proceed with the marriage regardless of the results of the premarital testing (32). In our study, 11.1% (44 participants) stated that they would get married even if both couples were carriers of the same disorder. The finding that a significant proportion of participants refused to change their decision despite both couples being carriers of the same condition would call for conducting qualitative research to determine the reasons for these decisions. One possible explanation is the test timing (33) as the premarital genetic screening test is usually done in the period just before the marriage. Moreover, couples or their families may ignore a positive result for various cultural, social, and emotional reasons (33). One possible solution is to perform the test during the high school or university stage, generally before 18 years. In contrast, in a study conducted in Saudi Arabia (34), 90 % of the couples at risk of having children affected with sickle cell disease or beta thalassemia still decided to marry because of fear of social stigma and because wedding plans could not be cancelled. A possible explanation for the relatively low percentage of participants who said that they would disregard the premarital test result (11.1%) in comparison with the later study in Saudi Arabia (90%) may be related to the fact that the investigation is not in a premarital context, so the question of what would they do is more a hypothetical one (since there is no social pressure on the participant to decide on the issue).

Even though we selected Qatari participants from different health centers in Qatar, we had a high response rate to ensure the generalization of results. However, our study has limitations, such as the cross-sectional design, which compromises the causality and temporality of associations between the variables. Moreover, the non-probability sampling method (convenience sampling) was used to collect the data, which hindered the generalizability of the results for the entire country.

## Conclusion

In summary, this study indicated that the prevalence of consanguineous marriage is high in Qatar, and most participants had a positive perception of consanguineous marriage. However, most of the participants needed to be made aware of the seriousness of the problem and its potential impact. Moreover, most of the study population did not hear about genetic blood diseases. These results could be changed by incorporating this information into the secondary school curriculum by raising awareness among adolescents and young adults about the negative impact of consanguineous marriage and the potential genetic problems in future offspring. Adolescents should also receive health education to change their attitudes and opinions, particularly before engagement. In addition, increasing the number of educational programs in media such as TV, radio, and newspapers is an option that should be considered for mass outreach. Finally, a qualitative study can be carried out among the Qatari population to better understand their perception and attitude toward consanguineous marriage.

## Data availability statement

The raw data supporting the conclusions of this article will be made available by the authors, without undue reservation.

## Ethics statement

The study involved human participants and was reviewed and approved by the Institutional Review Board (IRB) of Primary Health Care Corporation (PHCC/DCR/2022/06/032). The participants provided their written informed consent to participate in this study.

## Author contributions

YA: conceptualization, methodology, investigation, writing, reviewing and editing, formal analysis, and writing—original draft, data curation, and project administration. KA: conceptualization, methodology, and writing—reviewing and editing. MI: methodology, formal analysis, reviewing, and editing. MA, AA, and HA: methodology, writing—reviewing and editing, and data curation. NS and MY: methodology, writing—reviewing and editing, and supervision. All authors contributed to the article and approved the submitted version.
